# Intervention and Mechanisms of Alanyl-glutamine for Inflammation, Nutrition, and Enteropathy: A Randomized Controlled Trial

**DOI:** 10.1097/MPG.0000000000002834

**Published:** 2020-09

**Authors:** Sean R. Moore, Laura A. Quinn, Elizabeth A. Maier, Marjorie M. Guedes, Josiane S. Quetz, Madeline Perry, Chethan Ramprasad, Gabriela M.L. Lanzarini Lopes, Jordi Mayneris-Perxachs, Jonathan Swann, Alberto M. Soares, José Q. Filho, Francisco S. Junior, Alexandre Havt, Noelia L. Lima, Richard L. Guerrant, Aldo A.M. Lima

**Affiliations:** * Division of Pediatric Gastroenterology, Hepatology, & Nutrition, Department of Pediatrics, University of Virginia, Charlottesville, VA; † Division of Gastroenterology, Hepatology, & Nutrition, Department of Pediatrics, Cincinnati Children’s Hospital Medical Center, Cincinnati, OH; ‡ Department of Physiology and Pharmacology, Clinical Research Unit & Institute of Biomedicine/Center for Global Health, Faculty of Medicine, Federal University of Ceará, Fortaleza, Ceará, Brazil; § Department of Internal Medicine, NYU School of Medicine/NYU Langone Medical Center, New York, NY; ∥ Department of Emergency Medicine, Maine Medical Center, Portland, ME; ¶ Department of Diabetes, Endocrinology and Nutrition, Dr. Josep Trueta University Hospital, and Girona Biomedical Research Institute (IDIBGI), Girona, Spain.; # Center for Physiopathology of Obesity and Nutrition (CIBERobn), Instituto de Salud Carlos III, Madrid, Spain; ** Department of Metabolism, Digestion and Reproduction, Imperial College, London; †† School of Human Development and Health, Faculty of Medicine, University of Southampton, UK; ‡‡ Division of Infectious Disease and International Health, Department of Medicine, Center for Global Health, University of Virginia, Charlottesville, VA.

**Keywords:** barrier function, enteropathy, repair nutrients, stunting

## Abstract

**Objective:**

Determine the minimum dosage of alanyl-glutamine (Ala-Gln) required to improve gut integrity and growth in children at risk of environmental enteropathy (EE).

**Methods:**

This was a double-blinded randomized placebo-controlled dose-response trial. We enrolled 140 children residing in a low-income community in Fortaleza, Brazil. Participants were 2 to 60 months old and had weight-for-age (WAZ), height-for-age (HAZ), or weight-for-height (WHZ) *z*-scores less than −1. We randomized children to 10 days of nutritional supplementation: Ala-Gln at 3 g/day, Ala-Gln at 6 g/day, Ala-Gln at 12 g/day, or an isonitrogenous dose of glycine (Gly) placebo at 12.5 g/day. Our primary outcome was urinary lactulose-mannitol excretion testing. Secondary outcomes were anthropometry, fecal markers of inflammation, urine metabolic profiles, and malabsorption (spot fecal energy).

**Results:**

Of 140 children, 103 completed 120 days of follow-up (24% dropout). In the group receiving the highest dose of Ala-Gln, we detected a modest improvement in urinary lactulose excretion from 0.19% on day 1 to 0.17% on day 10 (*P*=0.05). We observed significant but transient improvements in WHZ at day 10 in 2 Ala-Gln groups, and in WHZ and WAZ in all Ala-Gln groups at day 30. We detected no effects on fecal inflammatory markers, diarrheal morbidity, or urine metabolic profiles; but did observe modest reductions in fecal energy and fecal lactoferrin in participants receiving Ala-Gln.

**Conclusions:**

Intermediate dose Ala-Gln promotes short-term improvement in gut integrity and ponderal growth in children at risk of EE. Lower doses produced improvements in ponderal growth in the absence of enhanced gut integrity.

Childhood undernutrition and diarrhea remain leading causes of death and disability in low- and middle-income countries (LMIC), unacceptably impeding progress towards 2030 global targets for childhood nutrition and survival (1). Environmental enteropathy (EE), a subclinical condition of small intestinal inflammation and barrier dysfunction, is highly prevalent in these settings and is hypothesized to be a gut manifestation of the reciprocal cycle between childhood undernutrition and diarrhea where undernutrition is both a risk factor for, and consequence of, diarrhea (2). Universal food security and adequate water, sanitation, and hygiene remain urgent unmet needs; however, adjunct interventions to prevent and reverse EE and mitigate chronic linear growth stunting are also needed (3).

Gut-trophic “repair nutrients” remain an area of intense interest in the search for adjunct therapies for EE (4). Glutamine is a key fuel for rapidly proliferating cells, including intestinal epithelial cells and lymphocytes (5). In addition to acting as a major precursor for protein synthesis, renal gluconeogenesis, and nucleic acid biosynthesis; glutamine also regulates metabolism, promotes cellular proliferation and antioxidant formation, and modulates cytokine production (6). It is postulated that during periods of severe physiologic stress, the demand for glutamine outpaces the rate of endogenous production making it “conditionally essential” (7). Furthermore, in-vitro and animal studies have supported the intriguing possibility that glutamine deficiency could underlie the failure of gut homeostasis that occurs during physiological stress (8,9). Oral supplementation with alanyl-glutamine (Ala-Gln; 24 g a day for 10 days) improved short-term gut integrity and weight velocity 4 months after therapy in a group of undernourished children from Northeast Brazil (10), and has also been shown to decrease the duration of acute diarrhea (11).

We designed the Intervention and Mechanisms of Alanyl-Glutamine for Inflammation, Nutrition, and Enteropathy (IMAGINE) study to address the following questions: what is the lowest dose of Ala-Gln that improves intestinal barrier function and nutritional status in children at risk of underweight, wasting, or stunting? 2) By what immune or metabolic mechanisms, does Ala-Gln exert these potential benefits?

## METHODS

### Study Type and Location

The IMAGINE Trial (NCT01832636) was a prospective randomized double-blinded placebo-controlled dose-response trial conducted in the Parque Universitário urban community in Fortaleza, Brazil. A detailed description of this population is available in previous publications (10). The protocol and consent forms for this study were approved by the institutional review board at the Federal University of Ceara, Fortaleza, Brazil, and at the Cincinnati Children’s Hospital Medical Center. This work was funded by the Bill and Melinda Gates Foundation (grant OPP1066140 to R.L.G. and A.A.M.L.) and Fogarty International Center/NIH (K02TW008767 to S.R.M.).

### Selection and Enrollment of Participants

**FIGURE 1.**
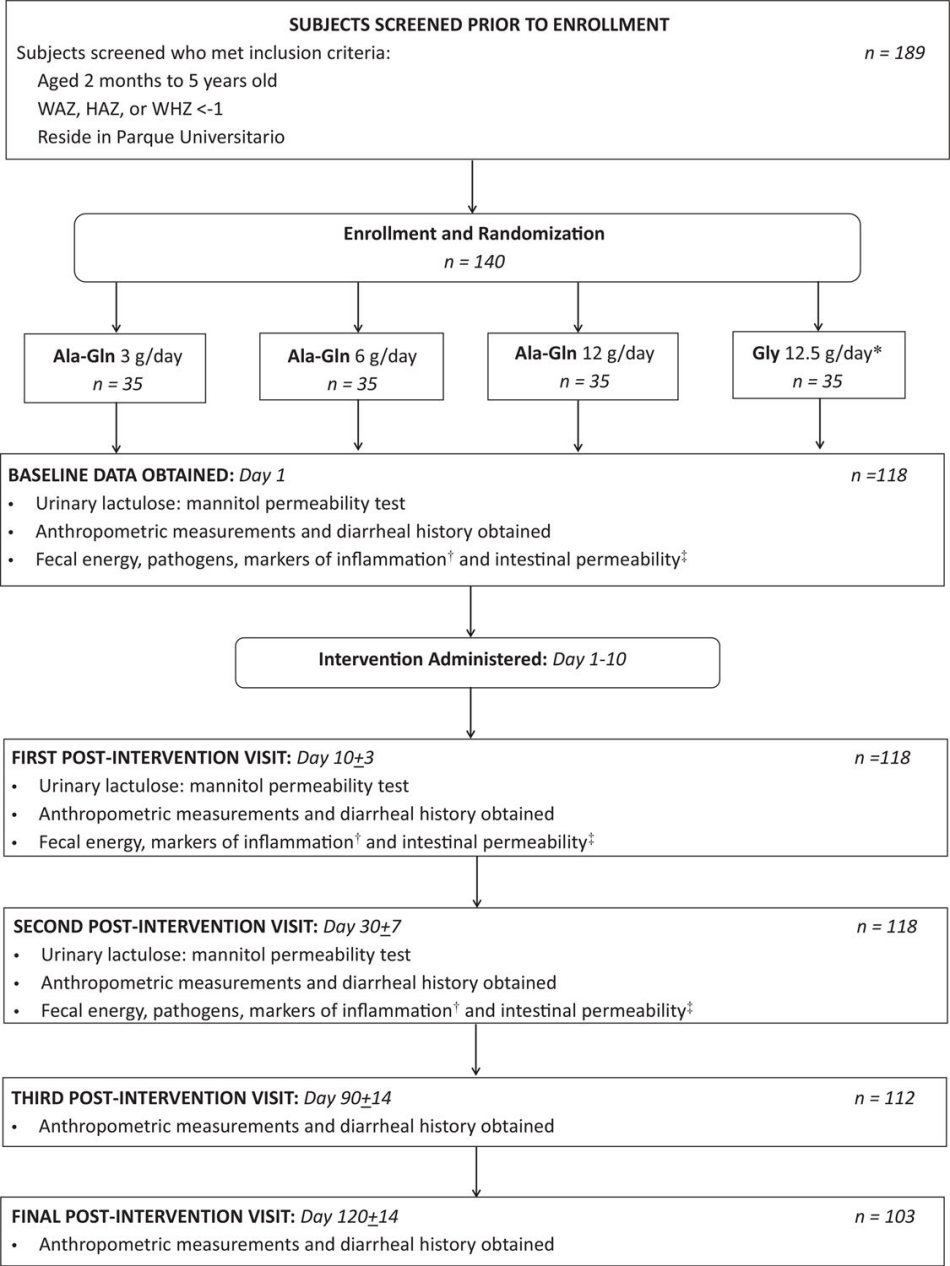
Flow diagram of study population and protocol. *The dosage of Gly placebo was isonitrogenous to the Ala-Gln 12 g/day group. †Markers of fecal inflammation: lactoferrin, myeloperoxidase, neopterin, Reg-1β, and fecal IL-2, IL-4, IL-6, IL-8, IL-10, GM-CSF, IFN-γ, TNF-α. ‡Markers of intestinal permeability: fecal alpha-1-antitrypsin. Ala-Gln = alanyl-glutamine.

Children ages 2 to 60 months old residing in the Parque Universitário neighborhood with HAZ, WAZ, or WHZ less than or equal to −1 were considered for inclusion. Those with clinical evidence of systemic disease, fever >38.8 °C, or antibiotic use at the time of screening were excluded, as were children with history of exclusive breast-feeding, chronic disease, previous participation in an intervention study, or those unable to ingest and retain nutritional supplements. Out of a desire to support exclusive breast-feeding per WHO guidelines, we targeted Ala-Gln as an intervention for children who are weaned or in the process of weaning from exclusive breast-feeding. Signed consent was obtained from a parent or guardian before enrollment. A total of 140 participants were enrolled between October 2013 and December 2015, and were followed for 120 days (Fig. 1).

### Intervention and Study Design

Using permuted block randomization, participants were assigned to receive 1 of 4 dietary supplements: Ala-Gln at 3 g/day (n=35); Ala-Gln at 6 g/day (n=35); Ala-Gln at 12 g/day (n=35); or glycine (Gly) at 12.5 g/day (n=35). Glycine, unlike glutamine, is not a preferential fuel for intestinal epithelial cells. Glycine was selected as a placebo to control for effects of dietary amino acid supplementation at a dose isonitrogenous to the highest dose of Ala-Gln. The Ala-Gln therapeutic supplement (Ajinomoto, Saõ Paulo, Brazil) or Gly placebo (Spectrum Chemical, Gardena, CA) was mixed with 50mL of milk, formula, or juice, and study personnel directly observed administration and ingestion. The study design is illustrated in Figure 1. Participants and study personnel were blinded to treatment allocation.

### Measures

#### Intestinal Permeability Test

Participants over 10 kg ingested a solution of 5 g of lactulose (Duphar Laboratories, Southampton, UK) and 1 g of mannitol (Henrifarma Produtos Químicos e Farmacêuticos Ltda, Saõ Paulo, Brazil) dissolved in 20mL of water after at least 3 hours of fasting. Children under 10 kg received 2 mL/kg of the solution. Urine was collected over the following 5 hours and 0.236 mg/mL of chlorhexidine (Sigma Chemical, St Louis, MO) was added before storage at −20°C. Percent excretion of lactulose and mannitol was measured by high-performance liquid chromatography with pulsed amperometric detection (12).

#### Fecal Biomarkers

Enzyme-linked immunosorbent assays were carried out to detect the following fecal measures: alpha-1-antitrypsin (A1AT; BioVendor, Candler, NC) as a measure of fecal protein loss and intestinal permeability, myeloperoxidase (MPO; Immundiagnostick, Bensheim, Germany) as a marker of intestinal mucosal neutrophil activity, neopterin (NEO; Benway Biotech, San Diego, CA) as a marker of T-helper cell 1 activity, and Reg-1β (TechLab, Blacksburg, VA) as a measure of intestinal epithelial regeneration. Specimens were stored at −20°C before analysis. After thawing at room temperature, they were diluted in a buffer with protease inhibitors and analyzed via standard curves provided by the assay manufacturers. Fecal lactoferrin (LFF) was used as a marker of intestinal inflammation and was measured via agglutination assay using the LUEKO-TEST kit from TechLab (Blacksburg, VA) using previously described methods (13).

#### Fecal Cytokine Measurement

Fecal samples (2.5 g) were initially diluted with a 5.0mL protease inhibitor solution containing 100mL of 1×PBS, 100 mg of phenylmethylsulfonyl fluoride and 100 mg of soy trypsin inhibitor (Sigma, EUA). This solution was centrifuged at 10,000 *g* for 15 minutes at 4 °C then passed through a 0.45mm filter. Fecal cytokines (IL-2, IL-4, IL-6, IL-8, IL-10, GM-CSF, IFN-γ, TNF-α) were measured using the Bio-Plex Pro Human Cytokine 8-plex Assay and the Bio-Plex Manager software was used for data acquisition and analysis (Bio-Rad Laboratories, Hercules, CA).

#### Fecal Calorimetry

Approximately 25 g of feces from a single stool was collected and stored at −80 °C (14). Specimens were thawed at 4 °C for 24 hours, homogenized, frozen at −80 °C, and lyophilized (Labconco Freeze Dryer). A 0.1 to 0.3 g aliquot of dried stool was pelletpressed, spiked with a benzoic acid tablet of known energy content, and bomb calorimetry was performed in duplicate according to the manufacturer’s instructions (Oxygen Bomb Calorimetry-Parr Instrument Company) (15).

See Supplemental Digital Content 1 (http://links.lww.com/MPG/B879) for methods for anthropometric measurements, urine metabolomics, stool microbiology, diarrheal morbidity, and adverse events.

### Sample Size and Statistical Analysis

As an objective measure of overall intestinal barrier function, the lactulose-mannitol test was selected as the primary outcome variable and was used to calculate the sample size. To detect a 30% reduction in lactulose-mannitol ratios with an alpha level of *P*=0.05 and 90% power, we calculated a requirement of 22 participants in each experimental group. This calculation was based on data from preliminary studies in the same population (lactulosemannitol ratio=0.13±0.04). A total of 140 participants, or 35 participants per group were enrolled to account for a possible dropout rate of 30%.

Analyses were conducted on an intention-to-treat basis using GraphPad Prism (version 8.3.0, GraphPad Prism, La Jolla, CA) and the open-source WHO *igrowup* R package. Means (Ms) and standard deviations (SDs) are reported for normally distributed data and medians (Mdn) with interquartile ranges (IQR) for nonnormally distributed data. Lactulose-mannitol excretion tests and fecal markers were log-transformed before analysis. Unpaired *t*-tests and Mann-Whitney *U*- tests were used to analyze normally distributed or nonparametric data, respectively. Chi-squared tests were used to compare the prevalence of abnormal results per group over time. A *P*-value <0.05 was considered significant.

## RESULTS

Of 140 children, 104 (74%) completed all 3 postintervention visits. A total of 36 participants did not complete the full protocol: 22 before day 1; 6 between 30 and 90 days; and 9 between 90 and 120 days. Reasons for noncompletion included change of address (9/140, 6%), voluntary dropout (3/140, 2%), and discontinuation for unknown reasons (4/140, 3%).

At study entry, there were no significant differences between groups in age, sex, anthropometric *z*-scores, diarrheal history, or ULM ratios, which were within the range of values for healthy children in this community (Table 1) (12). Further, no baseline differences were found in the number of abnormal stools, fecal markers and cytokines, or fecal energy between groups (Table, Supplemental Digital Content 2, http://links.lww.com/MPG/B879). At enrollment, 19.5% (n=128) of patients had abnormal testing for stool parasites including *Giardia lamblia* (10.1%), *Ascaris lumbricoides* (5.5%), *Entamoeba coli* (3.1%), *Cryptosporidium* (2.3%), *Trichomonas* (2.3%), and *Enterobius vermicularis* (1.5%). Only 2 patients had evidence of 2 concomitant stool parasites. Although fecal markers of inflammation and fecal cytokines were within the range previously reported in healthy controls, fecal calorimetry was slightly elevated compared with previously reported normal values with M (SD) of 5143.2 (505.5) (16).

**FIGURE 2.**
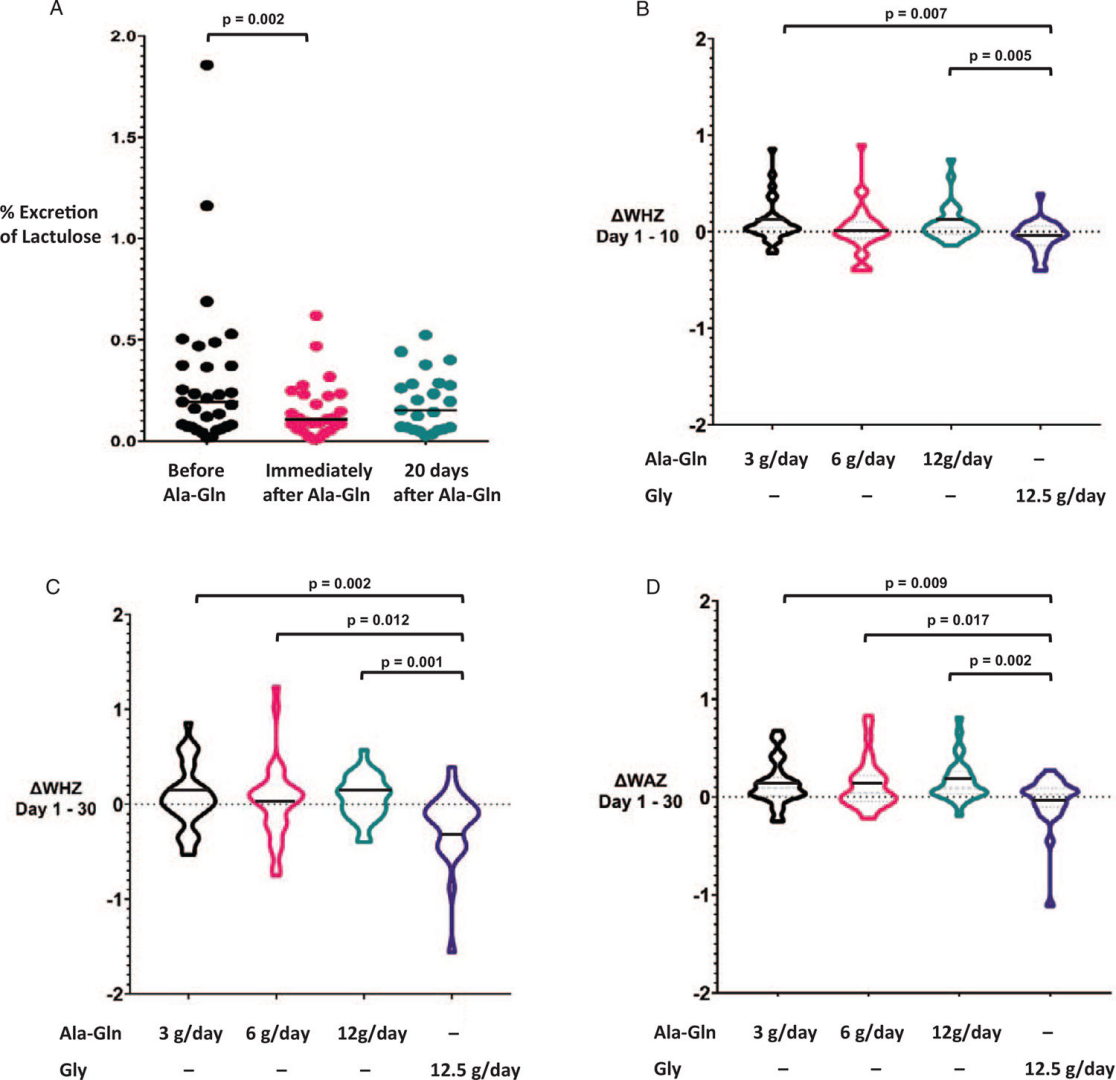
Intermediate dose Ala-Gln (12 g/day) supplementation improves percent lactulose excretion, WHZ, and WAZ in children at risk of environmental enteropathy. (A) Percent lactulose excretion in children treated with Ala-Gln 12 g/day, solid lines represent medians. (B) Effect of Ala-Gln on cumulative WHZ between days 1 and 10, (C) effect of Ala-Gln on cumulative WHZ between days 1 and 30, (D) effect of Ala-Gln on cumulative WAZ between days 1 and 30. In (B), (C), and (D), violin plots depict data distribution and density, solid lines represent means. Ala-Gln = alanyl-glutamine.

In the Ala-Gln 12 g/day (intermediate dose) group, we detected a significant decrease in percent lactulose excretion between day 1 and 10 (Fig. 2), suggesting enhanced barrier function. This improvement in gut integrity did not persist at day 30 (Table 2). Improvements in lactulose excretion were neither observed at lower doses of Ala-Gln nor in the placebo group. The ULM ratio and the percent excretion of mannitol did not significantly change in treatment groups or the control group over time (Table 2).

A significant improvement in WHZ was noted in the Ala-Gln 3 g/day (ΔWHZ=0.095) and Ala-Gln 12 g/day (ΔWHZ=0.09) groups at day 10 when compared with Gly (ΔWHZ=−0.054). At day 30, a significant improvement in both WHZ (Ala-Gln 3 g/day ΔWHZ=0.074, Ala-Gln 6 g/day ΔWHZ=0.074, Ala-Gln 12 g/day ΔWHZ=0.072, Gly ΔWHZ=−0.230) and WAZ (Ala-Gln 3 g/day DWAZ=0.134, Ala-Gln 6 g/day DWAZ=0.128, Ala-Gln 12 g/day DWAZ=0.166, Gly DWAZ=−0.04) was seen in all treatment groups. This improvement, however, did not persist at day 90 or 120 (Fig. 2; Table, Supplemental Digital Content 3, http://links.lww.com/MPG/B879).

As an increase in ponderal growth at day 30 was observed in the absence of any improvement in barrier function, urine metabolic profiles were analyzed in the children randomized to Ala-Gln 6 g/day to explore the possibility that it was promoting weight gain through metabolic alterations. PCA and OPLS-DA models were constructed to compare the urinary metabolic profiles of children receiving Ala-Gln 6 g/day or the placebo Glycine at baseline and day 10 or 30. No clear between-group variation was, however, observed in either the PCA or OPLS-DA analyses (Figure, Supplemental Digital Content 4, http://links.lww.com/MPG/B879).

There was a trend towards improved absorption in the treatment groups that was not statistically significant. Over 10 days, there was an increase in fecal energy (cumulative change 360 kcal/g±637, n=16) in the participants receiving Gly. Conversely, fecal energy decreased (cumulative change −82 kcal/g±624, n=14) in the participants receiving Ala-Gln 3 g/day. Although fecal energy increased in participants receiving 6 and 12 g/day of Ala-Gln (cumulative change 82±473, n=18; 114±1237, n=12), these were relatively modest when compared with the Gly group (Table, Supplemental Digital Content 2 and 5, http://links.lww.com/MPG/B879).

Fecal markers of inflammation and cytokine profiles were obtained on days 1 (n=135) and 10 (n=98). A significant (*P*=0.0247) decrease in lactoferrin was observed between day 1 (Mdn 2.1 μg/g, IQR 1.1–5.5) and day 10 (Mdn 0.8 μg/g, IQR 0.4–3.9) in the group receiving Ala-Gln 3 g/day. No changes were noted in fecal A1AT, MPO, NEO, Reg-1B, or cytokines between treatment groups or over time (Table, Supplemental Digital Content 6, http://links.lww.com/MPG/B879). The percentage of participants found to have enteric parasites significantly increased (*P*=0.028) from 19.5% (n=128) to 33.7% (n=74) on day 30, with *Giardia* accounting for the majority of this increase (day 1=10%, day 30=20%, *χ*^2^=0.042). Interestingly, Ala-Gln was associated with increasing *Giardia* prevalence (*χ*^2^ test for trend, day 1–30, *P*=0.04) but Gly was not. Despite this, there was no significant difference in parasite prevalence between groups at any point and a stratified analysis did not show any effect of parasite presence on urinary lactulose-mannitol excretion testing or anthropometry. Fecal GM-CSF was elevated in participants with parasites (*P*<0.0001).

A total of 34 (28.8%) adverse events (AEs) occurred in the 118 participants. There were no significant AEs (SAEs) resulting in death, threat to life, hospitalization, or significant disability or incapacity (10). The most common AE was fever (n=14, 11.8%), followed by cough (n=4, 3.4%), impetigo (n=3, 2.5%), and vomiting (n=3, 2.5%). There was no significant difference in the number or type of AEs between groups (Table, Supplemental Digital Content 7, http://links.lww.com/MPG/B879).

## DISCUSSION

The goal of our study was to determine the minimum dose at which Ala-Gln improves barrier function and nutritional status in children in Northeastern Brazil at risk for EE, and to clarify the mechanism through which it exerts these benefits. In this study, we found that 12 g/day of Ala-Gln improved intestinal barrier function as measured by urinary lactulose, which is in agreement with previous work in this patient population using a higher dose of 24 g/day (10). At this lower dose, however, we did not detect an improvement in barrier function persisting beyond 10 days. Interestingly, we found a significant improvement in ponderal growth in children receiving lower doses of Ala-Gln in the absence of any improvements in barrier function.

As the positive impact on barrier function and anthropometric measures observed did not persist at later time points, our results suggest that a higher dose or more prolonged course of glutamine might be required to produce a robust and sustained improvement in intestinal barrier function in children with EE. With the exception of our earlier study (10), which used a dose of 24 g/day for 10 days (>1 g/kg), the majority of studies in children used a lower dosing range (0.3–0.6 · gkg^−1^ ··day^−1^) and found no improvements in barrier function (11,17–19). Dosing regimens in adult studies have ranged from 24 to 44 g/day (0.3–0.6 · gkg^−1^ · day^−1^) (20–22). It is plausible that growing children at risk of undernutrition might require a higher relative dose. Another important question raised by our findings is durability of gut integrity and ponderal growth effects after cessation of glutamine supplementation. Previous studies have utilized months-long dosing periods (19); however, to our knowledge, only 1 reported anthropometric measures after cessation of supplementation (23) and none have assessed durability of improvements in barrier function.

In considering trials of higher doses or longer periods of supplementation, safety considerations are important. Although early studies suggested that parenteral glutamine was of benefit to postoperative or trauma patients (24,25), it was associated with increased mortality in a large study of adult ICU patients with multiorgan failure (26). Nevertheless, numerous trials investigating enteral supplementation in children demonstrated no serious adverse effects (10,11,17–19,23,27–29). Interestingly, this study is the first to track or identify increases in parasite burden after glutamine supplementation. An abundance of Ala-Gln could aid *Giardia* in establishing colonization through a number of potential pathways. Glutamine is a precursor for arginine, which is the main fuel source for Giardia, and also an effector of Giardia-induced intestinal epithelial cell quiescence (30). Alanine is one of Giardia’s most abundant intracellular amino acids; it is secreted in response to hypoosmolar environments via antiport exchange with other aminoacids including L-glutamine (31). It is important to note that our subgroup analysis did neither suggest that increased parasite burden blunted Ala-Gln efficacy, nor was it associated with increased morbidity or decreased tolerability.

There is robust in-vitro evidence that glutamine modulates intercellular tight junctions and can reverse experimentally induced increases in intestinal cell permeability (32–34). In this study, it is possible that the lactulose-mannitol tests failed to fully capture the gut health benefits of glutamine. Dual sugar absorption tests are the most validated and widely used in-vivo assays of intestinal barrier function, and they correlate with current and future anthropometry in the large multinational MAL-ED study (35,36). Reference values are heterogeneous; and however, the assay fluctuates in response to exercise, illness, and dietary changes (37–39). Further standardization and validation of the lactulose-mannitol test, including age-specific standards, will facilitate comparison of findings and better establish the responsiveness of the test to intervention (40).

Our results suggest glutamine has a positive, albeit transient, effect on ponderal growth through mechanisms independent of improvement in intestinal barrier function. Previous clinical trials of glutamine supplementation are mixed, with some showing an improvement in weight gain (11,20,41) and others demonstrating no benefit (19,21). In vivo studies have probed numerous potential mechanisms including increased triglyceride absorption (42), sodium and water absorption (43), and increased protein synthesis rates (44). These gains are not attributable solely to extracellular fluid gain or increased adiposity. In children newly diagnosed with acute lymphoblastic leukemia, significant improvements in prealbumin and retinol-binding protein as well as decreased levels of edema were noted after 4 weeks of glutamine (29). Similarly, in adults with AIDS and unintentional weight loss, an over 4-fold increase in metabolically active tissue measured by bioelectrical impedance was noted after 12 weeks of glutamine (20).

Although we observed an intriguing relative decrease in fecal energy in patients receiving Ala-Gln versus placebo, we cannot confidently attribute superior ponderal growth in the treatment groups to enhanced intestinal absorption. Quantifying energy absorption in an LMIC community setting is challenging, and the multiday long collection periods standardly employed for metabolic balance studies would have been difficult for a study of this size. To our knowledge, utilization of spot fecal calorimetry in children with EE is unprecedented and limited in its inability to account for variation in meals, fecal energy, or stool volume. In a comparison of cystic fibrosis patients, a spot fecal calorimetry measurement was comparable with a 72-hour collection method (14).

The search for repair nutrients to curtail stunting and its sequelae remains open to the imagination. EE is thought to result from interactions of marginal diets and heightened exposure to gut pathogens early in life leading to a maladaptive cycle of dysbiosis, inflammation, and impaired permeability and absorption. Such stresses can lead to metabolic derangements with implications for growth, development, and health. For example, children from a single community with either Kwashiorkor, marasmus, stunting, or normal growth have unique metabolic phenotypes (45), and metabolomic signatures early in life predict neurocognitive and growth outcomes (46). An ideal nutritional intervention would be personalized to meet the biochemical demands of the individual metabolic system and would target a number of the aberrations underlying EE, making combination supplementation a focus of interest (4). Given its potential in supporting ponderal growth, Ala-Gln could be a promising component of adjunct therapies.

The IMAGINE study was limited by several factors. First, we did not include a 24 g/day dose of Ala-Gln arm that would have allowed us to make direct comparisons with our earlier trial. Second, the participants we recruited were less stunted and had lower lactulose excretion than those recruited using similar criteria in previous trials from the same population (10). Hence, less baseline enteropathy may have masked response to Ala-Gln supplementation. Third, the median age of our participants at enrollment was 31 months, beyond the critical period when most linear growth faltering is reversible. Given that our study was designed to detect a change in urinary lactulose-mannitol, it may have been inadequately powered to detect differences in secondary outcomes. Lastly, the fact that the study was carried out in a single community limits the generalizability of these findings.

## CONCLUSIONS

In conclusion, intermediate and lower doses of Ala-Gln promote transient improvements in ponderal growth in children at risk for EE in the absence of a consistent measurable improvement in intestinal barrier function. Despite having assessed a wide span of measures, we were unable to identify a single overriding mechanism through which Ala-Gln exerts this benefit, but would posit that it may modulate inflammation or absorption. Future studies should elucidate the relationship between Ala-Gln and *Giardia* infection, the durability of Ala-Gln-mediated improvements in barrier function or ponderal growth, effects in populations with a higher burden of EE and stunting, and combination therapies addressing pathogen burden and inflammation.

## Supplementary Material

Supplemental material

## Figures and Tables

**TABLE 1.** Baseline demographic characteristics of enrolled participants

		Study groups			
Parameters	Total	Ala-Gln 3g/day n=35	Ala-Gln 6g/day n=35	Ala-Gln 12g/day n=35	Gly 12g/day n=35
Age, months (mean ± SD)	31.4±16.98	28.3±15.22	31.8±16.25	32.7±19.36	33.5±17.43
Sex					
Male, n (%)	70 (50%)	21 (60%)	19 (54%)	14 (40%)	16 (46%)
Anthropometry (mean ± SD)					
WAZ	−0.358±0.978	−0.172±1.050	−0.522±0.922	−0.564±0.811	−0.325±1.104
HAZ	−1.72±0.720	−1.623±0.587	−1.820±0.700	−1.902±0.614	−1.802±0.614
WHZ	0.830±1.309	0.941±1.532	0.649±1.336	0.728±0.995	0.953±1.460
History of diarrhea, n (%)	1 (0.8%)	0 (0%)	0 (0%)	0 (0%)	1 (3.8%)
Lactulose:mannitol ratio, median (IQR)	0.049 (0.027–0.095)	0.056 (0.027–0.091)	0.032 (0.026–0.056)	0.067 (0.038–0.150)	0.048 (0.028–0.102)

Ala-Gln = alanyl-glutamine; HAZ = height-for-age; SD = standard deviation; WAZ = weight-for-age; WHZ = weight-for-height.

**TABLE 2.** Intestinal permeability parameters by time and group

Intestinal permeability measurements by time	Study groups*			
	Ala-Gln, 3 g/day	Ala-Gln, 6 g/day	Ala-Gln, 12 g/day	Gly, 12.5 g/day
Lactulose to mannitol ratio, median (IQR)				
Day 1	0.056 (0.027–0.091)	0.032 (0.026–0.056)	0.067 (0.038–0.150)	0.048 (0.028–0.102)
Day 10±3	0.057 (0.022–0.107)	0.031 (0.024–0.070)	0.058 (0.029–0.100)	0.064 (0.032–0.079)
Day 30±7	0.046 (0.030–0.123)	0.042 (0.032–0.083)	0.044 (0.030–0.080)	0.045 (0.031–0.068)
Percent excretion of mannitol, median (IQR)				
Day 1	3.257 (2.279–6.216)	3.709 (1.656–7.421)	2.787 (0.716–6.300)	2.833 (0.666–6.009)
Day 10±3	5.737 (2.169–8.853)	3.210 (1.341–7.025)	2.301 (0.627–4.856)	2.556 (1.585–4.484)
Day 30±7	3.267 (1.745–5.105)	2.734 (1.925–5.335)	3.249 (1.691–5.073)	1.784 (0.929–4.356)
% excretion of Lactulose, median (IQR)				
Day 1	0.201 (0.087–0.301)	0.123 (0.083–0.246)	0.193 (0.072–0.373)^†^	0.160 (0.073–0.210)
Day 10±3	0.205 (0.091–0.409)	0.131 (0.075–0.215)	0.107 (0.068–0.226)^†^	0.167 (0.075–0.246)
Day 30±7	0.198 (0.105–0.272)	0.148 (0.084–0.308)	0.152 (0.064–0.278)	0.098 (0.074–0.199)

Ala-Gln = alanyl-glutamine; IQR = interquartile range.

* Sample sizes by group (day 1, day 10, day 30): Ala-Gln 3 g/day (n_1_=30, n_10_=23, n_30_=25), Ala-Gln 6 g/day (n_1_=28, n_10_=23, n_30_=24), Ala-Gln 12 g/day (n1=31, n_10_=27, n_30_=23), Gly (n_1_=30, n_10_=26, n_30_=20).

† Unpaired *t*-test, *P*=0.05.

## What Is Known

- Environmental enteropathy is a subclinical condition characterized by intestinal inflammation and barrier dysfunction that is implicated as a cause of oral vaccine failure, stunting, and impaired neurocognitive development in children in low- and middle-income countries.
- The nutritional supplement alanyl-glutamine has been shown to improve intestinal barrier function in children with environmental enteropathy when administered orally at high doses over brief supplementation periods.

## What Is New

- When administered at low doses to children at risk for environmental enteropathy, oral alanyl-glutamine improves ponderal growth in the absence of a measurable improvement intestinal barrier function. At intermediate doses, it both improves barrier function and ponderal growth.
- The duration of effect of low and intermediate dose alanyl-glutamine is days to months.
