# Supplementary material for: Intervention and Mechanisms of Alanyl-glutamine for Inflammation, Nutrition, and Enteropathy: A Randomized Controlled Trial
Source: J Pediatr Gastroenterol Nutr. Author manuscript; Available in PMC 2021 Nov 9. (PMC8576339; doi:10.1097/MPG.0000000000002834)
Supplement: Supplemental material [file NIHMS1713576-supplement-Supplemental_material.pdf]

Supplemental Digital Content 1

Anthropometric Measurements

Trained investigators received quarterly refresher training on measuring height and weight. Supine height was obtained for participants under the age of 24 months and standing height was obtained for patients over the age of 24 months with scale accuracy of 0.1 cm. Body weight was measured on a calibrated digital scale (Tanita Solar Scale, Arlington, IL) with an accuracy of 100 g.

Diarrhea Morbidity

The presence of diarrheal illness was assessed at baseline, on a daily basis during intervention administration, and at each post-intervention follow-up. Diarrhea was defined as 3 or more liquid stools occurring in the previous 24 hours. A day of diarrhea was defined as 3 or more looser than normal bowel movements in a 24-hour period, with distinct episodes separated by at least 2 days without diarrhea. Duration of episodes was classified as acute (< 7 days), prolonged acute (>6 and <14 days), or persistent (>13 days)[49].

Adverse Events:

Participants were monitored for adverse events at each time point. Adverse events (AE) were defined as any untoward medical event in a participant regardless of causal relationship with the intervention. Serious adverse events (SAE) were defined as any untoward medical event occurring in a participant resulting in death, threat to life, hospitalization, or significant disability or incapacity [10].

Microbiology:

Stool samples were examined for helminths and protozoa microscopic testing via previously described methods [47,48]. Briefly, formalin-ethyl acetate sedimentation was used to separate parasites from fecal debris (FPC Fecal Parasite Concentration Kit, Evergreen Scientific, Los Angeles, California). Organisms were identified morphologically by wet prep and modified acid-fast stain.

Urine metabolomics:

Urine samples were analyzed using ^{1}H nuclear magnetic resonance (^{1}H-NMR) spectroscopy via previously described methods (see Supplemental Digital Content for detailed methods) [50]. In brief, urine samples were mixed with phosphate buffer containing a 3-trimethylsilyl-1-[2,2,3,3,-$^{2}H_{4}$] propionate standard and centrifuged prior to spectroscopic analysis using a 700-MHz Bruker spectrometer equipped with a cryoprobe. For each sample, 8 dummy scans were followed by 128 scans collected into 64K data points. The mixing time was 10 ms, the acquisition time was 3.8 s, and the recycle delay was 2s. Spectra were manually phased and corrected for baseline distortions. Chemical shifts were normalized to the TSP peak at $\delta = 0.0$. Spectra were digitized using an in-house MATLAB script and were analyzed using principal components analysis (PCA) and orthogonal projection to latent structures discriminant analysis (OPLS-DA).

SUPPLEMENTARY DIGITAL CONTENT 2: Baseline fecal measures

Parameters	Total N=135	Study Groups: Ala-Gln 3 g/day n=35	Ala-Gln 6 g/day n= 33	Ala-Gln 12 g/day n=33	Gly 12.5 g/day n=34
Stool pathogen: n (%)	27 (26.7%)	7 (21.9%)	8 (26.7%)	3 (8.8%)	9 (29%)
Fecal markers: median (IQR)					
A1AT (ug/ml)	50.7 (9.8-119.3)	38.9 (7.1-102.5)	68.6 (13.5-173.1)	46.8 (18.2-100)	54.6 (18.3-100.4)
Reg-1B (ug/g)	79.1 (58.9-179.6)	89.8 (67.4-196.2)	71.8 (55.9-162.1)	68.2 (58-168.5)	79.1 (62.1-169.3)
LFF (ug/ml)	1.9 (0.6-5.6)	2.1 (1.1-5.5)	2.1 (0.8-5.5)	1 (0.4-5.6)	2 (0.7-5.9)
MPO (ng/ml)	435 (248-1551)	271 (191-905)	375 (244-2649)	698 (258-1919)	875 (248-1549)
NEO (nmol/L)	0 (0-0.3)	0 (0-0.3)	0 (0-11.1)	0 (0-0)	0 (0-5.1)
Fecal Cytokines: median (IQR) *					
IL-2 (pg/ml)	0.56 (0-1.18)	0.15 (0-0.89)	0 (0-1.53)	0.68 (0.2-2.56)	0.57 (0-1.38)
IL-4 (pg/ml)	0.02 (0-21)	0.02 (0-0.18)	0.07 (0-0.28)	0 (0-0.19)	0.03 (0-0.2)
IL-6 (pg/ml)	0.29 (0-0.69)	0.29 (0-0.58)	0.23 (0-0.53)	0.52 (0.23-1.05)	0.31 (0.03-0.68)
IL-8 (pg/ml)	2.81 (1.17-9.29)	1.55 (1.1-8.85)	3.6 (1.26-10.73)	1.71 (1.15-5.48)	4.4 (1.94-10.76)
IL-10 (pg/ml)	1.57 (1.3-2.26)	1.51 (1.21-2.11)	1.72 (1.42-2.02)	1.61 (1.29-2.35)	1.61 (1.29-2.72)
GM-CSF (pg/ml)	133.5 (76.4-325.1)	126.6 (70.7-351)	92.8 (67.1-188.4)	148.5 (96-296.3)	144.6 (96.3-296.3)
IFN-γ (pg/ml)	0 (0-7.01)	0 (0-4.64)	0 (0-2.79)	1.4 (0-13.86)	0 (0-6.59)
TNF-α (pg/ml)	0.75 (0.1-1.88)	0.64 (0.05-1.32)	0.86 (0.13-1.83)	0.81 (0.13-2.39)	0.81 (0.1-1.88)
Fecal calorimetry, kcal/g (mean ± SD)†	5.14 ± 0.5	5.15 ± 0.56	5.06 ± 0.3	5.33 ± 0.51	5.01 ± 0.57

* $N=134$, $n_{Ala\text{-}Gln\ 3\ g/day}=35$, $n_{Ala\text{-}Gln\ 6\ g/day}= 31$, $n_{Ala\text{-}Gln\ 12\ g/day}= 36$, $n_{Gly\ 12.5\ g/day}=32$

† $N=111$, $n_{Ala\text{-}Gln\ 3\ g/day}=27$, $n_{Ala\text{-}Gln\ 6\ g/day}= 27$, $n_{Ala\text{-}Gln\ 12\ g/day}= 29$, $n_{Gly\ 12.5\ g/day}=27$

SUPPLEMENTARY DIGITAL CONTENT 3: Cumulative anthropometric z-scores over 120-day follow-up

	Study groups							
	Ala-Gln 3 g/day		Ala-Gln 6 g/day		Ala-Gln 12g/day		Gly 12.5 day	
Delta cumulative z-scores	n	Mean ± SD	n	Mean ± SD	n	Mean ± SD	n	Mean ± SD
Weight-for-age								
1-10 days	32	0.080 ± 0.166	31	0.032 ± 0.186	29	0.081 ± 0.169	25	-0.014 ± 0.104
1-30 days	32	0.134 ± 0.225[*]	31	0.128 ± 0.253[†]	29	0.166 ± 0.217[‡]	26	-0.040 ± 0.266[*†‡]
1-90 days	29	0.096 ± 0.309	27	0.178 ± 0.422	28	0.062 ± 0.248	25	0.094 ± 0.306
1-120 days	24	0.195 ± 0.415	25	0.333 ± 0.458	28	0.299 ± 0.529	23	0.078 ± 0.566
Height-for-age								
1-10 days	32	0.017 ± 0.079	31	0.029 ± 0.078	29	0.018 ± 0.082	25	0.036 ± 0.080
1-30 days	32	0.154 ± 0.232	31	0.188 ± 0.247	29	0.164 ± 0.256	26	0.206 ± 0.245
1-90 days	29	0.204 ± 0.371	27	0.211 ± 0.327	28	0.156 ± 0.415	25	0.233 ± 0.252
1-120 days	24	0.181 ± 0.481	25	0.300 ± 0.372	28	0.477 ± 0.795	23	0.519 ± 0.520
Weight-for-height								
1-10 days	32	0.095 ± 0.221[§]	31	0.229 ± 0.253	29	0.090 ± 0.192[ǀ]	25	-0.054 ± 0.168[§ǀ]
1-30 days	32	0.074 ± 0.336[Φ]	31	0.040 ± 0.408 [Φ]	29	0.072 ± 0.228[Ψ]	26	-0.230 ± 0.379 [¶ΦΨ]
1-90 days	29	0.052 ± 0.467	27	0.131 ± 0.674	28	-0.009 ± 0.424	25	-0.156 ± 0.525
1-120 days	24	0.136 ± 0.625	25	0.226 ± 0.226	28	0.065 ± 0.630	23	-0.339 ± 0.884

WAZ 1-30: [*]p= 0.009, 95% CI [-0.303 to -0,045]; [†]p=0.017, 95% CI [-0.307 to -0.030]; [‡]p=0.002, 95% CI [-0.336 to -0.075]
WHZ 1-10: [§]p=0.007, 95% CI [-0.255 to -0.042]; [ǀ]p=0.005, 95% CI [-0.243 to -0.045]
WHZ 1-30: [¶]p=0.002, 95% CI [-0.492 to -0.116]; [Φ]p=0.012, 95% CI [-0.480 to -0.059]; [Ψ]p=0.001, 95% CI [-0.469 to -0.134]

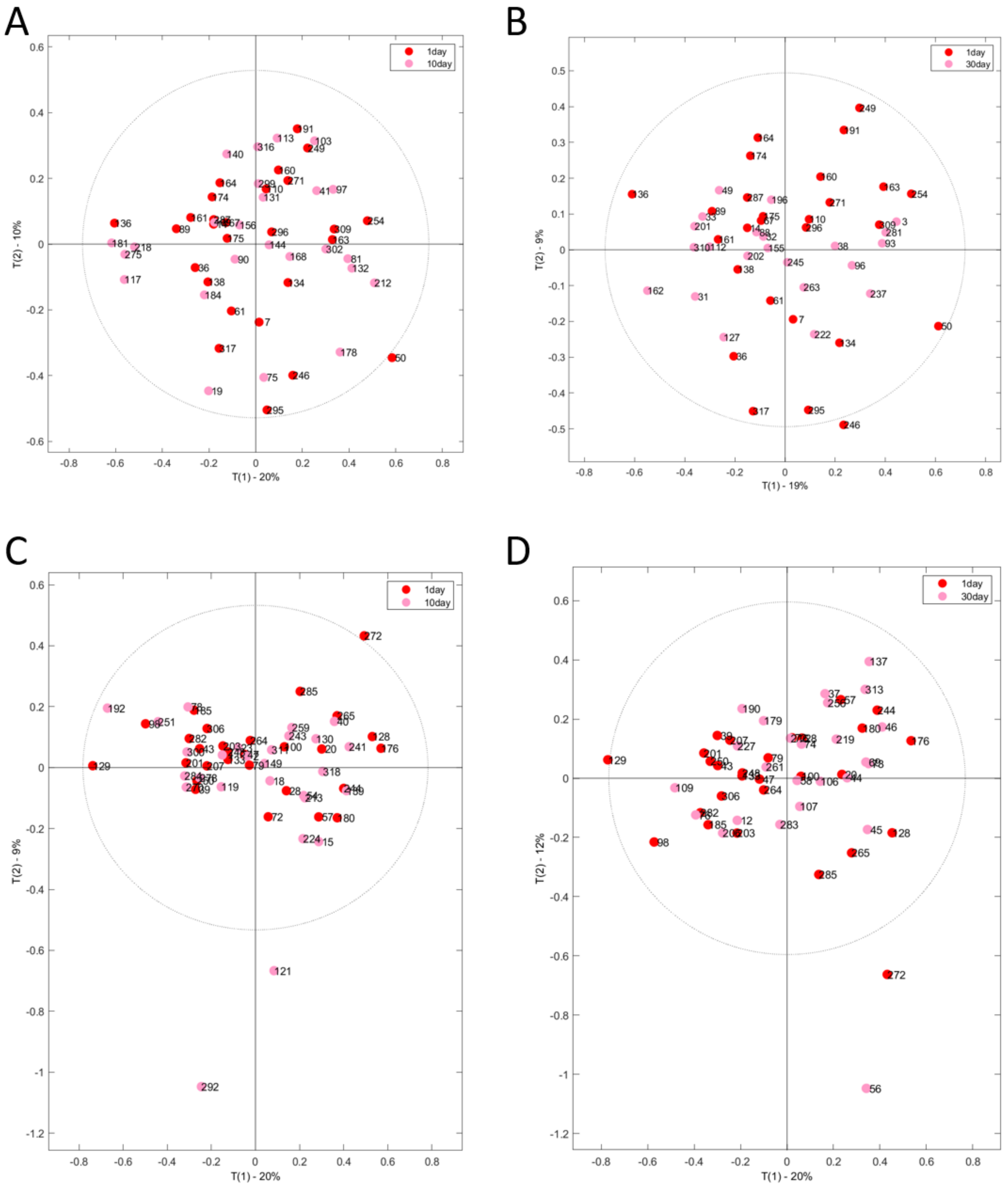

Figure, Supplemental Digital Content 4. PCA scores plots derived from the ^{1}H NMR spectra of urine from patients receiving Ala-Gln 6 g/day (A, B) and the placebo Glycine (C, D). The models compare the metabolic profiles obtained at baseline and day 10 (A, C) and baseline and day 30 (B, D). The PCA scores plots display baseline values in red and post-intervention values in pink. The ellipse represents the Hotelling's T2 95% confidence interval.

SUPPLEMENTARY DIGITAL CONTENT 5: Post-intervention fecal measures (day 10)

		Study Groups			
Parameters	Total	Ala-Gln 3 g/day n=25	Ala-Gln 6 g/day n= 27	Ala-Gln 12 g/day n=20	Gly 12.5 g/day n=26
Stool pathogen: n (%)	27 (31.4%)	7 (33.3%)	4 (17.4%)	7 (33.3%)	9 (39.1%)
Fecal markers: median (IQR)					
A1AT (ug/ml)	69.4 (21.1-115)	85.8 (34.4-118)	48.1 (12.6-104.3)	72.3 (35.5-115.7)	78.2 (21-122.5)
Reg-1B (ug/g)	83.4 (68.2-134.9)	89.8 (70.3-179.9)	81.2 (67.7-140.8)	84 (67.2-172.4)	76.9 (64.1-94.1)
LFF (ug/ml)	0.9 (0.5-3.5)	0.8 (0.4-3.9)	0.8 (0.3-3.1)	1 (0.6-3.7)	1.2 (0.6-2.9)
MPO (ng/ml)	348 (264-2003)	348 (271-1910)	287 (256-1219)	824 (248-2537)	459 (279-2003)
NEO (nmol/L)	0 (0-0)	0 (0-0)	0 (0-0)	0 (0-0.1)	0 (0-0)
Fecal Cytokines: median (IQR)*					
IL-2 (pg/ml)	0.33 (0-0.8)	0.21 (0-0.96)	0.14 (0-0.64)	0.39 (0-0.8)	0.56 (0-0.16)
IL-4 (pg/ml)	0.03 (0-0.17)	0 (0-0.11)	0 (0-0.18)	0.07 (0-0.19)	0.03 (0-4.4)
IL-6 (pg/ml)	0.29 (0-0.52)	0.29 (0-0.61)	0.24 (0-0.42)	0.42 (0-0.61)	0.29 (0-0.52)
IL-8 (pg/ml)	2.02 (0.99-5.86)	1.42 (0.88-5.86)	2.07 (1.04-6.59)	1.91 (1.44-3.63)	2.07 (0.9-6.62)
IL-10 (pg/ml)	1.77 (1.3-1.98)	1.57 (1.17-1.98)	1.78 (1.27-1.97)	1.77 (1.34-2.12)	1.87 (1.42-2.12)
GM-CSF (pg/ml)	132 (71.6-247.5)	126.6 (40.3-465.7)	92.8 (0-427.4)	148.4 (13.9-997)	144.6 (53.5-1100.7)
IFN-γ (pg/ml)	0 (0-3.63)	0 (0-2.79)	0 (0-0)	0 (0-8.98)	0 (0-9.52)
TNF-α (pg/ml)	0.75 (0.13-1.44)	0.86 (0.13-1.44)	0.75 (0.12-1.15)	0.86 (0.13-2.66)	0.75 (0.1-1.54)
Fecal calorimetry, kcal/g (mean ± SD) †	5.21 ± 0.62	5.14 ± 0.4	5.05 ± 0.34	5.51 ± 0.95	5.28 ± 0.66

* $n_{Ala\text{-}Gln\ 3\ g/day}=25$, $n_{Ala\text{-}Gln\ 6\ g/day}= 27$, $n_{Ala\text{-}Gln\ 12\ g/day}= 21$, $n_{Gly\ 12.5\ g/day}=23$

† $N=62$, $n_{Ala\text{-}Gln\ 3\ g/day}=15$, $n_{Ala\text{-}Gln\ 6\ g/day}= 18$, $n_{Ala\text{-}Gln\ 12\ g/day}= 12$, $n_{Gly\ 12.5\ g/day}=16$

SUPPLEMENTARY DIGITAL CONTENT 6: Adverse events (AEs) and serious adverse events (SAEs) by study group over 120-day follow-up

		Study Groups			
Parameters	Total n=118 (%)	AG 3 g/day n=32	AG 6 g/day n=31	AG 12g/day n=29	G 12.5 g/day n=26
AEs	34 (28.8)	5	10	9	10
Fever	14 (11.8)	4	3	3	4
Cough	4 (3.4)	1	2	1	0
Vomiting	3 (2.4)	1	1	0	1
Diarrhea	2 (1.7)	0	1	0	1
Impetigo	3 (2.5)	0	1	1	1
Urticaria	2 (1.7)	0	0	2	0
Allergic Reaction	1 (0.8)	0	0	1	0
Conjunctivitis	1 (0.8)	0	1	0	0
Chalazion	1 (0.8)	0	0	1	0
Poor Appetite	1 (0.8)	0	1	0	0
Dog Bite	1 (0.8)	0	0	0	1
Wound	1 (0.8)	0	0	0	1
SAEs	0	0	0	0	0
